# Hierarchical Superstructures by Combining Crystallization‐Driven and Molecular Self‐Assembly

**DOI:** 10.1002/anie.202105787

**Published:** 2021-06-17

**Authors:** Andreas Frank, Christian Hils, Melina Weber, Klaus Kreger, Holger Schmalz, Hans‐Werner Schmidt

**Affiliations:** ^1^ Macromolecular Chemistry I University of Bayreuth and Bavarian Polymer Institute Universitätsstrasse 30 95447 Bayreuth Germany; ^2^ Macromolecular Chemistry II University of Bayreuth and Bavarian Polymer Institute Keylab Synthesis and Molecular Characterization Universitätsstrasse 30 95447 Bayreuth Germany

**Keywords:** crystallization-driven self-assembly, hierarchical superstructures, molecular self-assembly, patchy polymer fibers, supramolecular structures

## Abstract

Combining the unique corona structure of worm‐like patchy micelles immobilized on a polymer fiber with the molecular self‐assembly of 1,3,5‐benzenetricarboxamides (BTAs) leads to hierarchical superstructures with a fir‐tree‐like morphology. For this purpose, worm‐like patchy micelles bearing pendant, functional tertiary amino groups in one of the corona patches were prepared by crystallization‐driven self‐assembly and immobilized on a supporting polystyrene fiber by coaxial electrospinning. The obtained patchy fibers were then immersed in an aqueous solution of a tertiary amino‐functionalized BTA to induce patch‐mediated molecular self‐assembly to well‐defined fir‐tree‐like superstructures upon solvent evaporation. Interestingly, defined superstructures are obtained only if the pendant functional groups in the surface patches match with the peripheral substituents of the BTA, which is attributed to a local increase in BTA concentration at the polymer fibers’ surface.

## Graphical abstract

Hierarchical fir‐tree‐like superstructures are accessible by utilizing the unique corona structure of patchy worm‐like micelles immobilized on electrospun polymer fibers and the molecular self‐assembly of a 1,3,5‐benzenetricarboxamide. The used combination of crystallization‐driven and molecular self‐assembly concepts can give access to a large variety of fibrillar superstructures with potential applications in filtration or catalysis.

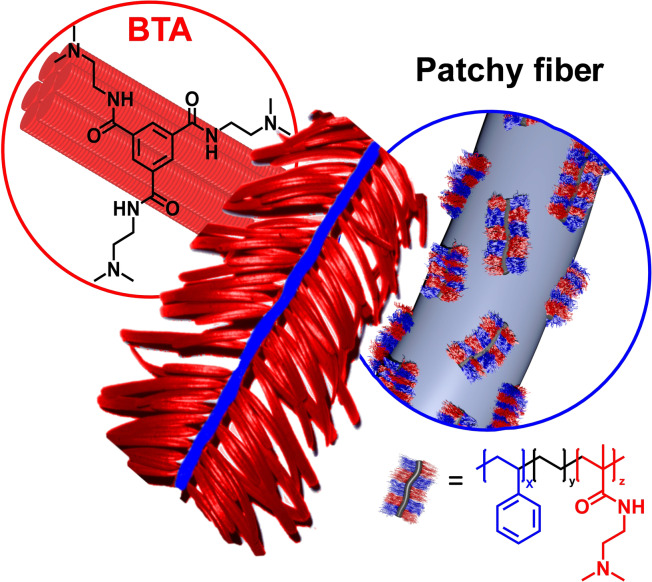

Hierarchical self‐assembly represents an intriguing approach to realizing complex superstructures at the nano‐ and mesoscale in nature. Although artificial molecular and macromolecular self‐assembly concepts have already proven to pave the way to more complex assemblies, the outstanding control over shape, dimension, and functionality found in nature is still far from being achieved. The guided hierarchical self‐assembly of amorphous block copolymers in solution has been shown to represent a facile way to tailor‐made multicompartment structures.^[1]^ However, particularly for one‐ (1D) and two‐dimensional (2D) structures, the precise control over size and size distribution is difficult to achieve. Herein, the introduction of a crystallizable block, which adds an additional and strong driving force for micelle formation, can be harnessed to solve these issues in a process termed crystallization‐driven self‐assembly (CDSA). Especially the discovery of living CDSA,^[2]^ which closely resembles the features of living polymerization processes with respect to control over size and size distribution, has given access to a multitude of complex and hierarchical micellar assemblies of controlled shape and size, like, for example, patchy and block comicelles, branched and scarf‐like micelles, colloidosomes and multidimensional superstructures.^[3]^ A concept closely related to CDSA is supramolecular polymerization, that is, the self‐assembly of small molecules via directed and reversible secondary interactions, which we denote as molecular self‐assembly in the following.^[4]^ Similarly, molecular self‐assembly processes allow the fabrication of defined nanostructures and superstructures.^[5]^ In particular, control over the self‐assembly process and, in turn, on the objects’ dimensions can be achieved with living supramolecular polymerization,^[6]^ which can be realized for instance by a seed‐initiated growth approach. The combination of a precise top‐down coating technique, such as coaxial electrospinning,^[7]^ with CDSA, a typical bottom‐up approach, was recently employed in our group to construct patchy hybrid nonwovens with excellent performance in heterogeneous catalysis.^[8]^ Utilizing a similar strategy for the immobilization of 1,3,5‐benzenetricarboxamide (BTA) seeds onto electrospun fibers yielded mesostructured nonwovens with a penguin downy‐feather‐like morphology by an in situ growth of supramolecular BTA fibers from the electrospun seed fibers.^[9]^ However, with this approach the control over the seed density on the electrospun fibers is limited.

Herein, we show that hierarchical fir‐tree‐like superstructures can be generated by the combination of two self‐assembly concepts, that is, crystallization‐driven self‐assembly and molecular self‐assembly (Scheme 1). Our conceptual approach to these defined superstructures comprises A) the preparation of patchy PS_core_/SEDMA fibers by coaxial electrospinning of polystyrene (PS) and patchy worm‐like polystyrene‐*block*‐polyethylene‐*block*‐poly(*N*,*N*‐dimethylaminoethyl methacrylamide) (SEDMA) triblock terpolymer micelles, B) the immersion of this patchy fibers into an aqueous *N* ^1^,*N* ^3^,*N* ^5^‐tris[2‐(dimethylamino)ethyl]‐1,3,5‐benzenetricarboxamide (BTA‐Methyl) solution and C) the controlled molecular self‐assembly of BTA‐Methyl to highly defined hierarchical superstructures upon solvent evaporation. In the resulting fir‐tree‐like superstructures the PS_core_/SEDMA fibers form the core, from which supramolecular BTA‐Methyl fibers (“needles”) have grown away from the surface in a well‐controlled fashion.

**Scheme 1:**
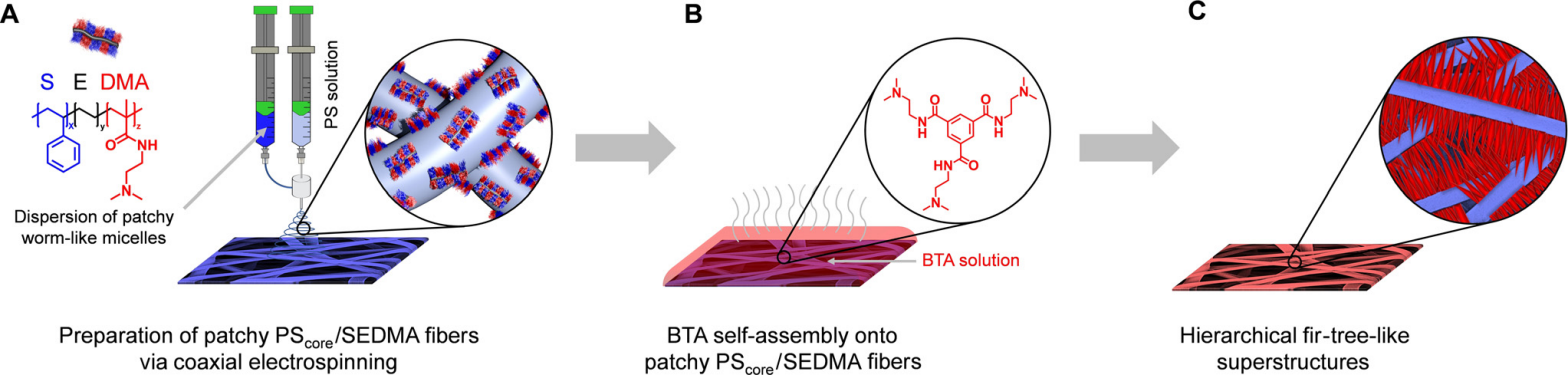
Formation of hierarchical fir‐tree‐like superstructures. A) Coaxial electrospinning of a polystyrene (PS) solution and a dispersion of patchy worm‐like polystyrene‐*block*‐polyethylene‐*block*‐poly(*N*,*N*‐dimethylaminoethyl methacrylamide) (SEDMA) triblock terpolymer micelles to prepare patchy PS_core_/SEDMA fibers. B) Immersion of the patchy PS_core_/SEDMA fibers into an aqueous *N* ^1^,*N* ^3^,*N* ^5^‐tris[2‐(dimethylamino)ethyl]‐1,3,5‐benzenetricarboxamide (BTA‐Methyl) solution and subsequent evaporation induced molecular self‐assembly of BTA‐Methyl onto the patchy fibers. C) Final hierarchical fir‐tree‐like superstructures after complete solvent evaporation.

The PS_core_/SEDMA fibers with functional surface patches were prepared by immobilizing patchy worm‐like SEDMA micelles on top of PS fibers by coaxial electrospinning, employing a PS solution (7 wt. % in DMF; *M*_n_=1.8×10^6^ g mol^−1^) as core and a dispersion of patchy worm‐like SEDMA micelles (*c=*10 g L^−1^ in THF) as shell, respectively (Scheme 1 A). The dispersion of the patchy worm‐like SEDMA micelles was prepared via CDSA, as previously reported, whereby the PS patches provide compatibility with the supporting PS fiber and the PDMA patches provide the functionality.^[10]^ Experimental details and the molecular characteristics of the employed triblock terpolymers are described in the Supporting Information. Exemplarily, a TEM image of the patchy worm‐like SEDMA micelles is depicted in Figure S1. Here, the semi‐crystalline PE block forms the core and the micellar corona consists of alternating, nanometer‐sized PS (18±5 nm) and PDMA (17±5 nm) patches (Table S1). Figure 1 A shows a scanning electron microscope image of a patchy PS_core_/SEDMA polymer fiber, where the worm‐like SEDMA micelles on top of the supporting PS fiber can be clearly distinguished.

**Figure 1:**
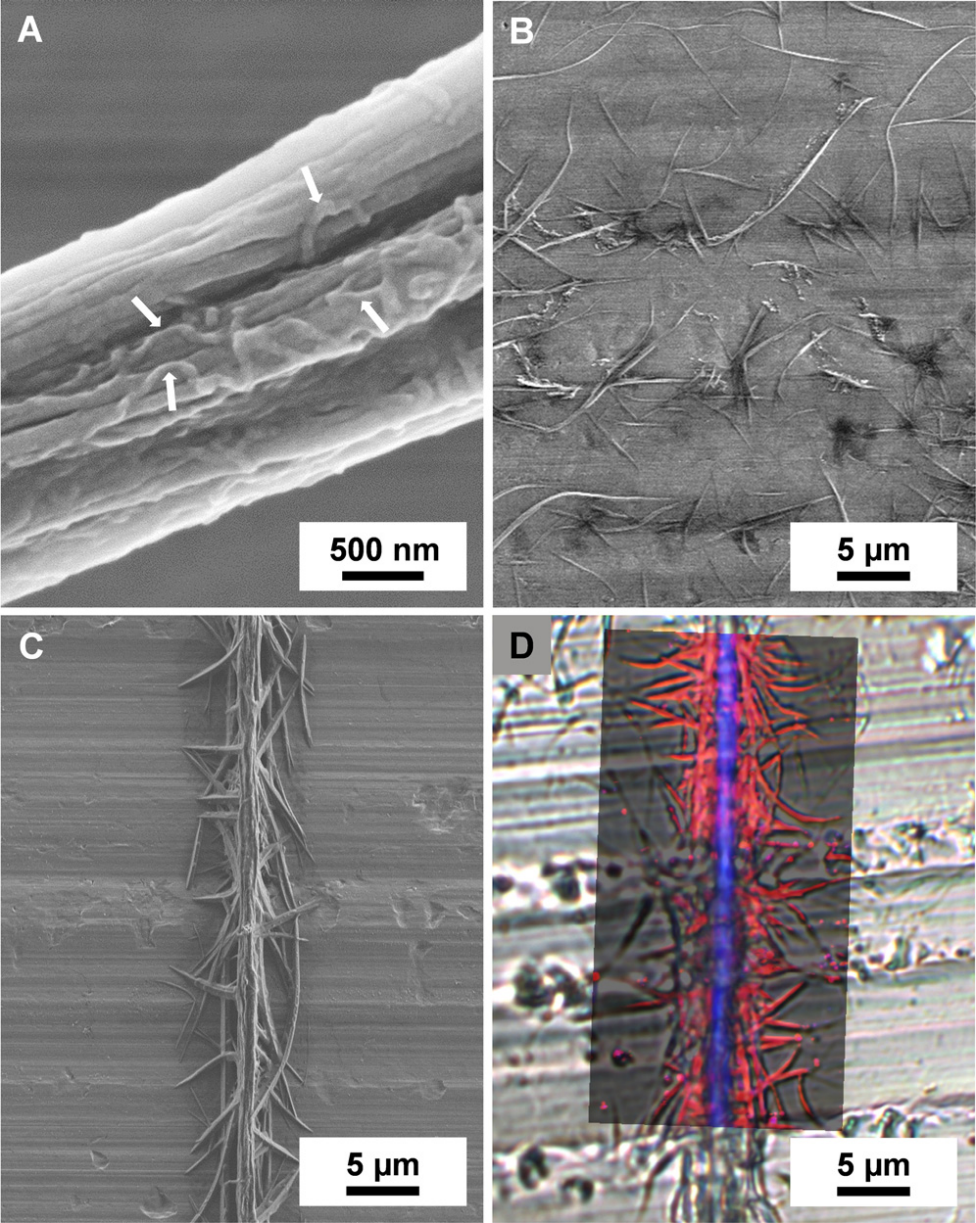
Scanning electron micrographs of A) a patchy PS_core_/SEDMA fiber (the arrows indicate the patchy worm‐like micelles on top of the supporting PS_core_ fiber); B) uncontrolled supramolecular BTA assemblies prepared upon solvent evaporation from a 0.050 wt. % aqueous solution on aluminum foil; C) a hierarchical fir‐tree‐like superstructure prepared by molecular self‐assembly of a 0.050 wt. % aqueous BTA‐Methyl solution onto patchy PS_core_/SEDMA fibers after solvent evaporation. D) Overlay of the spatially resolved component distribution from Raman imaging (PS‐rich regions are colored in blue and BTA‐Methyl rich in red, respectively) with the optical microscopy image of the fir‐tree‐like superstructure.

As building block for the molecular self‐assembly (Scheme 1 B) BTA‐Methyl was selected featuring peripheral tertiary *N*,*N*‐dimethylaminoethyl substituents^[11]^ which match the pendant functional groups in the PDMA patches of the SEDMA micelles. BTA‐Methyl is highly water soluble up to a concentration of 15 wt. %^[11]^ and exhibits no temperature‐induced formation of supramolecular structures at a concentration of 2 wt. %, as demonstrated by temperature‐dependent turbidity measurements and micro‐differential scanning calorimetry (Figure S2, S3). Only upon solvent evaporation a threshold concentration is apparently reached where homogeneous nucleation results in the formation of randomly distributed supramolecular BTA‐Methyl fibers with a broad length distribution (Figure 1 B). The final deposited fiber density correlates with the initially used concentration of BTA‐Methyl (Figure S4). In an analogous manner, also the PDMA block of the SEDMA triblock terpolymer shows complete solubility in water over a broad temperature range.^[12]^

Remarkably, immersion of the patchy PS_core_/SEDMA fibers into a BTA‐Methyl solution of only 0.05 wt. % followed by solvent evaporation (Scheme 1 B,C) resulted in highly defined hierarchical fir‐tree‐like superstructures as shown in Figure 1 C. Moreover, the BTA‐Methyl fibers grow away from the patchy polymer fiber in an oriented manner and can be observed at different positions, which indicates different starting points owing to the randomly distributed worm‐like micelles at the surface of the PS_core_/SEDMA fiber. This is confirmed by Raman imaging, which allows to distinguish between the patchy PS_core_/SEDMA fiber and the BTA fibers. The Raman spectra of the corresponding neat compounds can be found in Figure S5. Figure 1 D shows an overlay of the optical microscopy image of the fir‐tree‐like superstructure and the spatially resolved component distribution from Raman imaging (PS is depicted in blue and BTA‐Methyl in red), clearly demonstrating that the BTA‐Methyl fibers are directly attached onto the patchy PS_core_/SEDMA fiber. Furthermore, Raman imaging allows to probe the alignment of the BTA‐Methyl units, since the spectra differ depending on their orientation with respect to the polarization of the laser (horizontally polarized). Most of the BTA‐Methyl units show a perpendicular stacking to the PS fiber (Figure S6).

These results indicate that the patchy surface of the fibers is able to initiate the molecular self‐assembly of BTA‐Methyl. As PDMA is amorphous and well soluble in water, the PDMA patches are highly swollen and, accordingly, the pendant tertiary *N*,*N*‐dimethylaminoethyl groups are not expected to show any structural order. This makes a heterogeneous nucleation of BTA‐Methyl due to an epitaxial match highly unlikely. Hence, the nucleation effect might be attributed to an accumulation (local increase in concentration) of BTA‐Methyl in the PDMA patches at the fibers’ surface promoted by the chemical match of peripheral groups of BTA‐Methyl and pendant tertiary amino groups in the PDMA patches.

To get a closer insight into the superstructure formation via molecular self‐assembly of BTA‐Methyl mediated by the functional PDMA patches at the surface of PS_core_/SEDMA fibers, electrospun polymer fibers with immobilized, non‐functional patchy micelles (PS_core_/SEM) and neat PS fibers were prepared and used as reference (Figure 2). Here, SEM denotes patchy worm‐like micelles prepared by CDSA of a polystyrene‐*block*‐polyethylene‐*block*‐poly(methyl methacrylate) triblock terpolymer. Replacing the functional hydrophilic PDMA patches by hydrophobic poly(methyl methacrylate) (PMMA) patches in the micellar corona led predominantly to an accumulation of unstructured BTA‐Methyl assemblies near the polymer fiber, which is attributed to a drying effect (Figure 2 A). In a similar manner, neat PS fibers also resulted in non‐defined BTA structures near the polymer fibers, however to a slightly lesser extent (Figure 2 B). This clearly demonstrates that neither a neat PS fiber nor patchy SEM micelles on top of the supporting PS fiber are sufficient to initiate the molecular self‐assembly of BTA‐Methyl, underlining the necessity of a chemical match of the pendant tertiary amino groups in the micellar patches and the peripheral groups of BTA‐Methyl. Consequently, two decisive factors might be extracted from these observations: i) The patches need to be soluble in the solvent (here water) used for the molecular self‐assembly of BTA‐Methyl as, otherwise, they would not be accessible; and ii) hydrogen‐bonding and dipole interactions between the pendant tertiary amino groups in the functional PDMA patches and the peripheral substituents of BTA‐Methyl most likely result in a local increase of the BTA‐Methyl concentration within the PDMA patches. The latter might in turn facilitate the formation of nuclei and, thus, a patch‐mediated molecular self‐assembly of BTA‐Methyl from the surface of the patchy PS_core_/SEDMA fibers.

**Figure 2:**
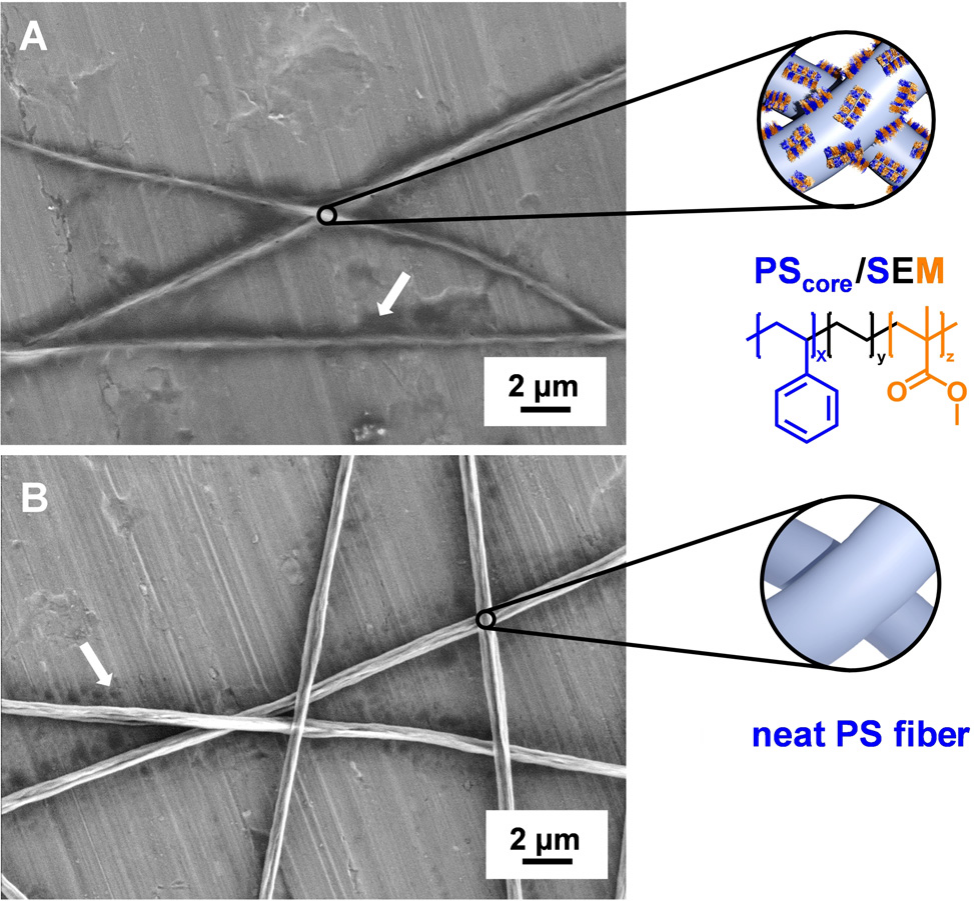
Scanning electron micrographs showing the absence of hierarchical superstructures for reference experiments employing A) PS fibers with patchy worm‐like micelles of polystyrene‐*block*‐polyethylene‐*block*‐poly(methyl methacrylate) (PS_core_/SEM) and B) neat PS fibers without patches in the molecular self‐assembly of BTA‐Methyl. The arrows indicate unstructured BTA‐Methyl assemblies formed upon drying.

To support our assumption that the PDMA patches locally increase the concentration of BTA‐Methyl and, thus, induce controlled self‐assembly, the concentration of the BTA‐Methyl solution was systematically varied (Figure 3). For a low BTA‐Methyl concentration of only *c=*0.025 wt. % in water, no molecular self‐assembly at the patchy PS_core_/SEDMA fibers was observed (Figure 3 A). Up to an initial concentration of *c=*0.100 wt. %, highly defined superstructures by patch‐mediated molecular self‐assembly were obtained (Figure 3 B,C). However, a further increase in concentration to *c=*0.250 wt. % led to very dense, less ordered supramolecular BTA‐Methyl fibers instead of well‐defined hierarchical superstructures (Figure 3 D). For the self‐assembly of neat BTA‐Methyl upon solvent evaporation, the formation of ordered structures was only observed from aqueous solutions with a concentration higher than *c=*0.250 wt. % (Figure S4). At this concentration, homogeneous nucleation is favored, resulting in star‐shaped assemblies where several fibers emanate from one central starting point. Consequently, there is a competition between the patch‐mediated molecular self‐assembly and homogeneous BTA‐Methyl nucleation. At low concentrations, that is, well below the concentration where self‐assembly is observed for neat BTA‐Methyl solutions, the patch‐mediated self‐assembly dominates and is driven by the local increase in BTA‐Methyl concentration in the PDMA patches allowing the formation of BTA‐Methyl nuclei. In contrast, at higher concentrations the homogeneous nucleation of BTA‐Methyl to randomly distributed supramolecular fibers is favored and control over the formed superstructure is lost.

**Figure 3:**
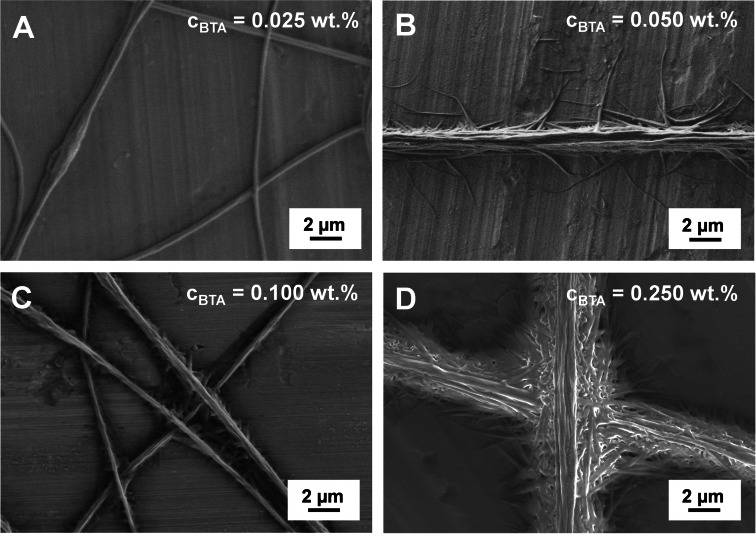
Scanning electron micrographs of hierarchical superstructures prepared by employing a A) 0.025 wt. %, B) 0.050 wt. %, C) 0.100 wt. % and D) 0.250 wt. % aqueous BTA‐Methyl solution for molecular self‐assembly onto patchy PS_core_/SEDMA fibers.

In conclusion, this work shows that polymer fibers with tailored functional surface patches can induce the molecular self‐assembly of a designed 1,3,5‐benzenetricarboxamide (BTA) into well‐defined hierarchical fir‐tree‐like superstructures. The key for the patch‐mediated molecular self‐assembly of the BTA from the polymer fibers is the chemical match of the functional groups in the surface patches and the BTA peripheral groups that in turn controls the solubility and accessibility of the patch for the BTA. In particular, a close chemical match between the patches and the periphery of the molecular building blocks and their concentration can be regarded as design criteria for such hierarchical superstructures. These results open the opportunity to construct a large variety of complex hierarchical superstructures, as the chemistry of the polymer patches and the BTA peripheral groups can be easily tuned to transfer this concept to other functional components and solvent systems. This might stimulate further research on the fabrication of functional hierarchical superstructures for (nano)particle separation and immobilization with potential applications in filtration and heterogeneous catalysis.

## Conflict of interest

The authors declare no conflict of interest.

## Supporting information

As a service to our authors and readers, this journal provides supporting information supplied by the authors. Such materials are peer reviewed and may be re‐organized for online delivery, but are not copy‐edited or typeset. Technical support issues arising from supporting information (other than missing files) should be addressed to the authors.

Supporting Information
