# Supplementary material for: Hierarchical Superstructures by Combining Crystallization‐Driven and Molecular Self‐Assembly
Source: Angew Chem Int Ed Engl. 2021 Jun 17;60(40):21767–71. doi: 10.1002/anie.202105787 (PMC8518951; doi:10.1002/anie.202105787)
Supplement: Supplementary file 1 — Supporting Information [file ANIE-60-21767-s001.pdf]

Angewandte Chemie
Eine Zeitschrift der Gesellschaft Deutscher Chemiker

Supporting Information

Hierarchical Superstructures by Combining Crystallization-Driven and Molecular Self-Assembly

Andreas Frank[+], Christian Hils[+], Melina Weber, Klaus Kreger, Holger Schmalz, and Hans-Werner Schmidt**

anie_202105787_sm_miscellaneous_information.pdf

Table of Contents

1. Experimental Procedures and Methods……………………………………………………………………………………………3

2. Supporting Figures………5

S1: Transmission electron micrograph of patchy worm-like SEDMA triblock terpolymer micelles…………………..……..5

S2: Temperature-dependent turbidity measurements of aqueous BTA-Methyl solutions…………………......................6

S3: Micro-differential scanning calorimetry measurements of aqueous BTA-Methyl solutions……………………………7

S4: Self-assembly of aqueous BTA-Methyl solutions upon solvent evaporation onto aluminium foil……………………..8

S5: Raman measurements……………………………………………………………………………………………...9

S6: Spatially resolved component distribution from Raman imaging……………………..………………………………..…9

3. References…….10

Experimental Procedures

Materials. All chemicals were used as received unless otherwise noted. *N*,*N*-dimethylformamide (DMF, 99%, Acros Organics), tetrahydrofuran (THF, ≥ 99.9%, VWR). Polystyrene (PS) was synthesized by anionic polymerization in THF at -80 °C using *sec*-butyllithium as initiator (M_n = 1.8·10^6 g·mol^{-1}, $Đ$ = 1.08).

Synthesis of N^1,N^3,N^5-tris[2-(dimethylamino)-ethyl]-1,3,5-benzenetricarboxamide (BTA-Methyl). BTA-Methyl was synthesized as described previously.[1] Briefly, trimesic acid trimethyl ester was dispersed in *N*,*N*-dimethylethylenediamine under an argon atmosphere. The mixture was heated to 125 °C, stirred overnight and subsequently allowed to cool down to room temperature. The resulting mixture was dispersed in acetone and heated until an almost clear solution was obtained. The hot solution was filtrated using a sintered glass funnel filter. The solvent was removed and the product was dried in a vacuum oven at 50 °C over night, yielding a white powder.

Synthesis of triblock terpolymers. Polystyrene-*block*-polyethylene-*block*-poly(methyl methacrylate) ($S_{40}E_{21}M_{39}^{108}$) was synthesized by a combination of living anionic polymerization and catalytic hydrogenation, as described elsewhere.[2] Polystyrene-*block*-polyethylene-*block*-poly(*N*,*N*-dimethylaminoethyl methacrylamide) ($S_{33}E_{17}DMA_{50}^{132}$) was prepared *via* post-polymerization functionalization of the poly(methyl methacrylate) (PMMA) block of $S_{40}E_{21}M_{39}^{108}$.[3,4] In the used triblock terpolymer notation the subscripts describe the mass fraction of the corresponding block in wt.% and the superscript denotes the overall number average molecular weight (M_n) in kg·mol^{-1}. M_n was determined by a combination of MALDI-ToF MS (matrix-assisted laser desorption/ionization – time of flight mass spectrometry) and ^{1}H NMR (nuclear magnetic resonance) spectroscopy, employing the absolute M_n of the polystyrene precursor from MALDI-ToF for ^{1}H NMR signal calibration.

Formation of patchy worm-like triblock terpolymer micelles. The patchy worm-like micelles were prepared by crystallization-driven self-assembly (CDSA) of the triblock terpolymers $S_{40}E_{21}M_{39}^{108}$ and $S_{33}E_{17}DMA_{50}^{132}$ in THF according to our previous work.[2,3] The polymers were dissolved in THF (c = 10 g·L^{-1}) at 65 °C for 0.5 h using a thermostated shaker unit (HCL-MKR 13, Ditabis). The self-assembly process occurred by subsequently cooling to the crystallization temperature (T_c) of the polyethylene middle block (**Table S1**). The process was allowed to proceed for 24 h with 200 rpm resulting in in the respective patchy worm-like micelle dispersions.

Table S1. Properties of the used patchy worm-like micelles.

Triblock terpolymer[a]	T_c [°C][b]	patch size [nm][c]		length of micelles [nm][c]	Ref.
		S	M / DMA		
$S_{40}E_{21}M_{39}^{108}$	20	13 ± 4	13 ± 4	520 ± 140	[2,5]
$S_{33}E_{17}DMA_{50}^{132}$	21	18 ± 5	17 ± 5	510 ± 310	[3]

[a] Subscripts describe the mass fraction of the corresponding block in wt.% and the superscript denotes the overall molecular weight in kg·mol^{-1}.
[b] crystallization temperature of the PE block, c = 10 g·L^{-1} in THF.
[c] Average sizes ± standard deviation as determined by TEM image analysis of at least 100 micelles/patches.

Electrospinning. *Preparation of patchy polymer fibres PS_{core} / SEM and PS_{core} / SEDMA.* Patchy polymer fibres were produced by coaxial electrospinning, according to our previous work.[3,6] To this end, a 7 wt.% polystyrene (PS_{core}) (M_n = 1.8·10^6 g·mol^{-1}) solution in DMF was used as core and for the shell dispersions of patchy worm-like SEM or SEDMA micelles in THF (c = 10 g·L^{-1}) were employed. The fibres were spun on a collector placed at a distance of 20 cm from the coaxial needle (COAX_2DISP sealed coaxial needles, LINARI NanoTech, d_{core} = 0.51 mm and d_{shell} = 1.37 mm) at a temperature of 20.8 °C and a relative humidity of ca. 30%. For electrospinning, a high voltage of 11.4 kV at the needle and -1.0 kV at the collector were applied. The feed rate of the PS_{core} solution was 1.2 mL·h^{-1} and for the micellar dispersions 1.0 mL·h^{-1}. Neat polystyrene fibres were prepared as reference material in the same manner but without using the micellar dispersions.

Self-assembly of aqueous BTA-Methyl solutions upon solvent evaporation onto aluminium foil. 25 µL of an aqueous BTA-Methyl solution with concentrations ranging from 0.025 to 1.000 wt.% were dropped onto aluminium foil and the solvent was allowed to evaporate at ambient conditions. After solvent evaporation, turbid films were obtained and investigated by scanning electron microscopy.

Self-assembly of aqueous BTA-Methyl solutions onto patchy polymer fibres. The PS_{core} / SEDMA and PS_{core} / SEM fibres as well as the neat PS fibres on aluminium foil were immersed into BTA-Methyl solutions (varying in concentration ranging from 0.025 wt.% to 0.250 wt.% with a pH value from 7 to 11, respectively) for a fixed time of 30 s and allowed to dry at ambient conditions for complete solvent evaporation.

Methods

Scanning electron microscopy. For scanning electron microscopy measurements, a FEI Quanta FEG 250 scanning electron microscope (Thermo Fisher Scientific) equipped with a field emission gun was used. The untreated samples, i.e. without applying a sputter coating, were measured in the beam deceleration mode (only Figure 1A, 1C and 3B) or in the low vacuum mode. Measurements in the beam deceleration mode were conducted under high vacuum at an acceleration voltage of 6 kV. This mode was used to image surfaces at high magnification with a concentric back scattered (CBS) electron detector, which is insensitive to sample charging. Here, an additional negative voltage (bias, -4 kV) was applied to the stage. In this way, the primary electrons were decelerated to 2 kV when reaching the sample and interacted electrons were accelerated toward the CBS detector. The samples measured in the low vacuum mode (water pressure of 40 Pa in the sample chamber) were mounted on a sample holder using an adhesive graphite pad and were investigated with an acceleration voltage of 3 kV with a large-field (gaseous secondary electron) detector (LFD) for topographical details.

Transmission electron microscopy. The morphology of triblock terpolymer worm-like micelles were analysed by elastic bright-field transmission electron microscopy (TEM) on a Zeiss 922 Omega EFTEM (Zeiss NTS GmbH, Oberkochen, Germany). Zero-loss filtered images were recorded digitally on a bottom mounted CCD camera system (Ultrascan 1000, Gatan) at an acceleration voltage of 200 kV. The micrographs were processed with the digital imaging processing system of Gatan (Digital Micrograph 3.9 for GMS 1.4). The samples were diluted to c = 0.1 g·L^{-1} and a droplet was placed onto a carbon coated copper grid. The residual solvent was immediately blotted by filter paper, dried in a vacuum oven (20 mbar, room temperature) and stained with RuO_4 vapor (selective staining of PS). The average length and patch size were determined by measuring at least 100 micelles/patches using the software ImageJ.[7]

Raman imaging. A WITec alpha 300 RA+ imaging system, equipped with a UHTS 300 spectrometer and a back-illuminated Andor Newton 970 EMCCD camera, was employed for confocal Raman imaging. The measurements were conducted at an excitation wavelength of λ = 532 nm, using a laser power of 4 mW and an integration time of 0.6 s·pixel^{-1} (100x objective, NA = 0.9, step size 100 nm·pixel^{-1}, software WITec Control FIVE 5.3). All spectra were subjected to a cosmic ray removal routine and baseline correction. The spatial distribution of PS and BTA-Methyl was determined using the tool “true component analysis” in the WITec Project FIVE 5.3 software.

Crystallization system Crystal16. Temperature-dependent turbidity measurements of aqueous BTA-Methyl solutions were determined optically for eight concentrations in parallel at a wavelength of λ = 645 nm using the crystallization system Crystal16 (Technobis Crystallization Systems). Concentrations of 0.05, 0.10, 0.25, 0.50, 0.75, 1.00, 1.50 and 2.00 wt.% of BTA-Methyl in water were prepared at room temperature. The transmittance at λ = 645 nm of each sample was recorded for two cycles in the range of 5 to 90 °C. Each cycle consists of a heating and cooling step with a scanning rate of 0.5 K·min^{-1}.

Micro-differential scanning calorimetry. Micro-differential scanning calorimetry measurements were performed on a SETARAM Micro DSC III using sealed measuring cells ("batch cells", V = 1 mL, stainless steel) filled with about 0.7 mL of aqueous solution of the BTA-Methyl at a concentration of c = 0.05 or 2.00 wt.%. The measurements were carried out applying a heating and cooling rate of 0.5 K·min^{-1}. The reference cell was filled with an equal amount of water.

Supporting Figures

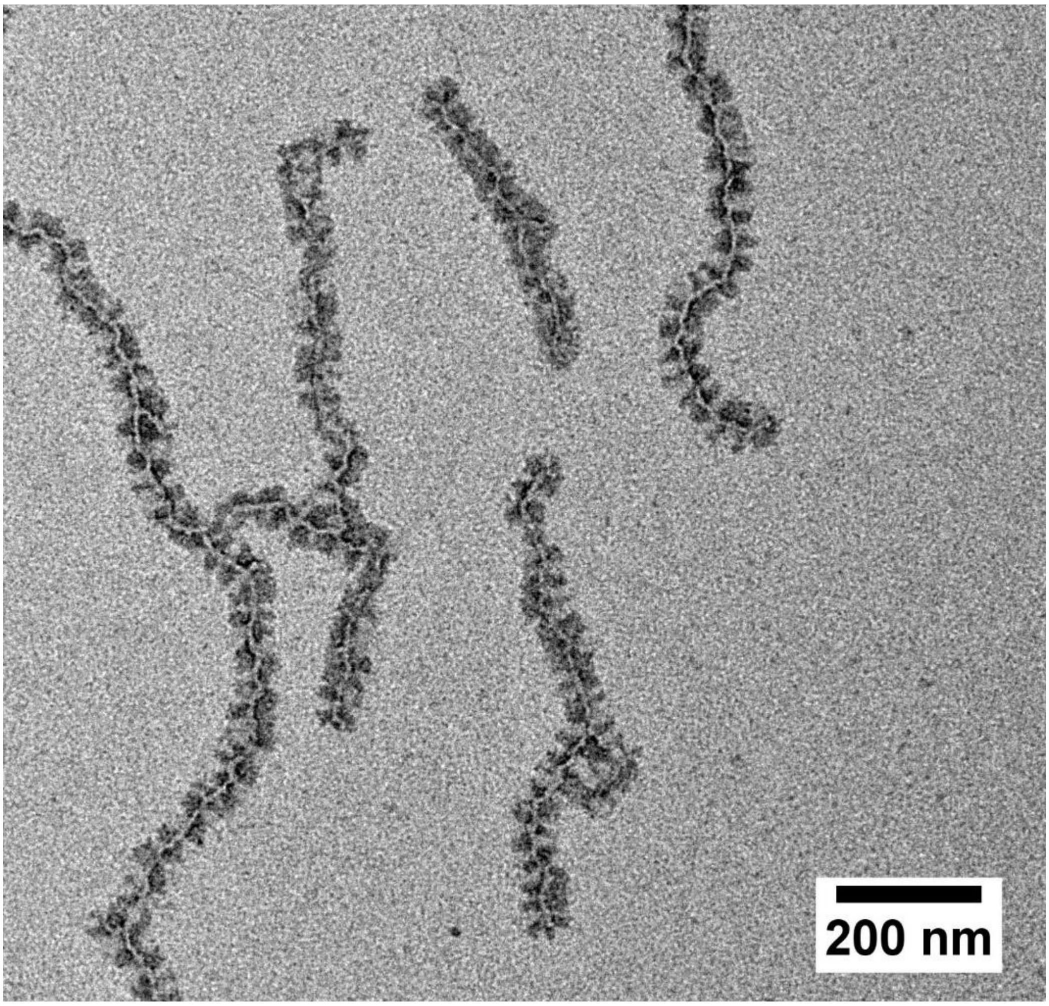

Figure S1. TEM micrograph of patchy worm-like $S_{33}E_{17}DMA_{50}^{132}$ micelles (c = 0.1 g·L^{-1} in THF). The micelles are composed of polyethylene (PE) as crystalline core and almost alternating corona patches of polystyrene (PS) and poly(*N*,*N*-dimethylaminoethyl methacrylamide) (PDMA). The polystyrene block was selectively stained with RuO_4 and appears dark.

Temperature-dependent turbidity measurement of aqueous BTA-Methyl solution. Exemplarily, both heating and cooling cycles and the recorded transmittance for BTA-Methyl with a concentration of *c* = 2.0 wt.% are shown in **Figure S2**. During heating and cooling all measured samples show a constant transmittance of 100% indicating that the BTA-Methyl remains dissolved during the measurements.

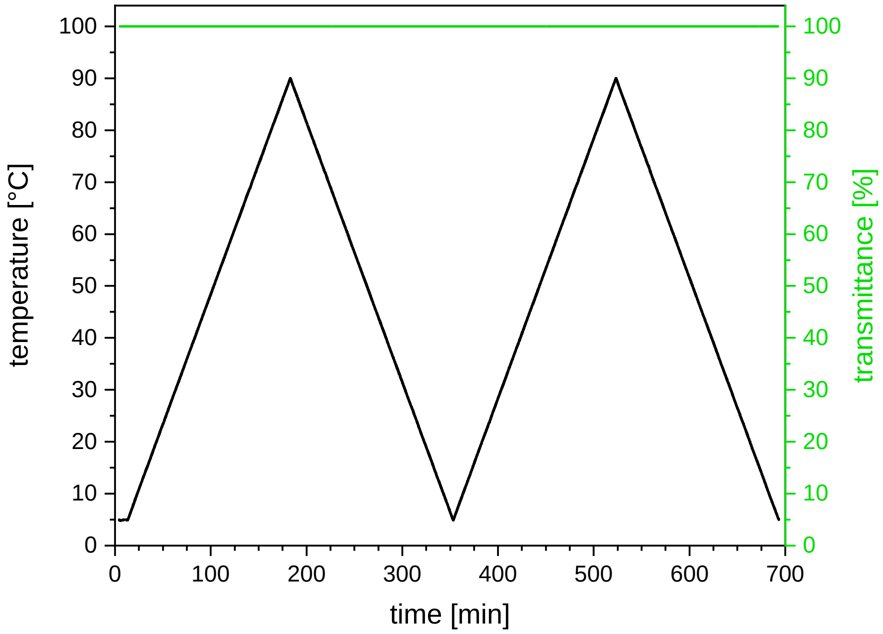

Figure S2. Temperature-dependent transmittance of a 2.00 wt.% aqueous BTA-Methyl solution upon subsequent heating and cooling cycles at a scanning rate of 0.5 K·min^{-1}.

Exemplarily, the 2nd heating and 2nd cooling traces for the aqueous BTA-Methyl solution with a concentration of c = 2.00 wt.% is shown in **Figure S3**. The same behaviour is observed for the 0.05 wt.% BTA-Methyl solution.

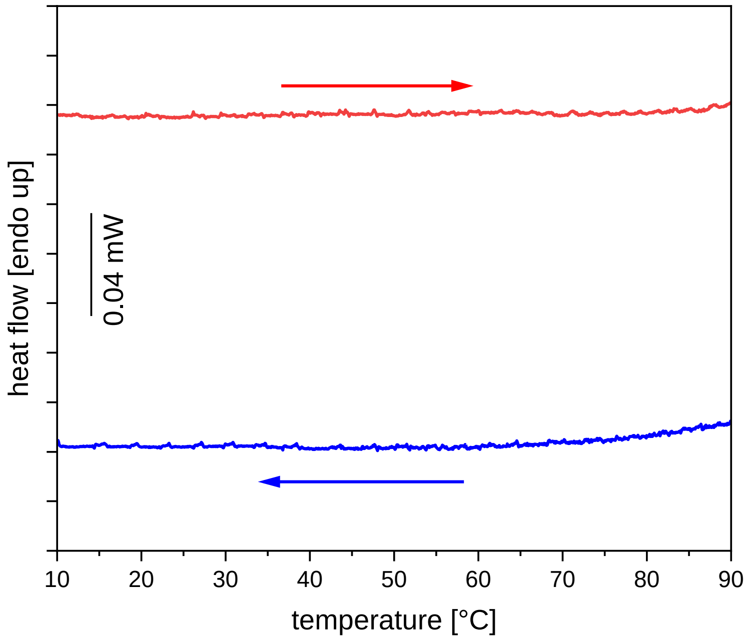

Figure S3. Micro-differential scanning calorimetry measurement of BTA-Methyl in water with a concentration of 2.00 wt.% at a scanning rate of 0.5 K·min^{-1}.

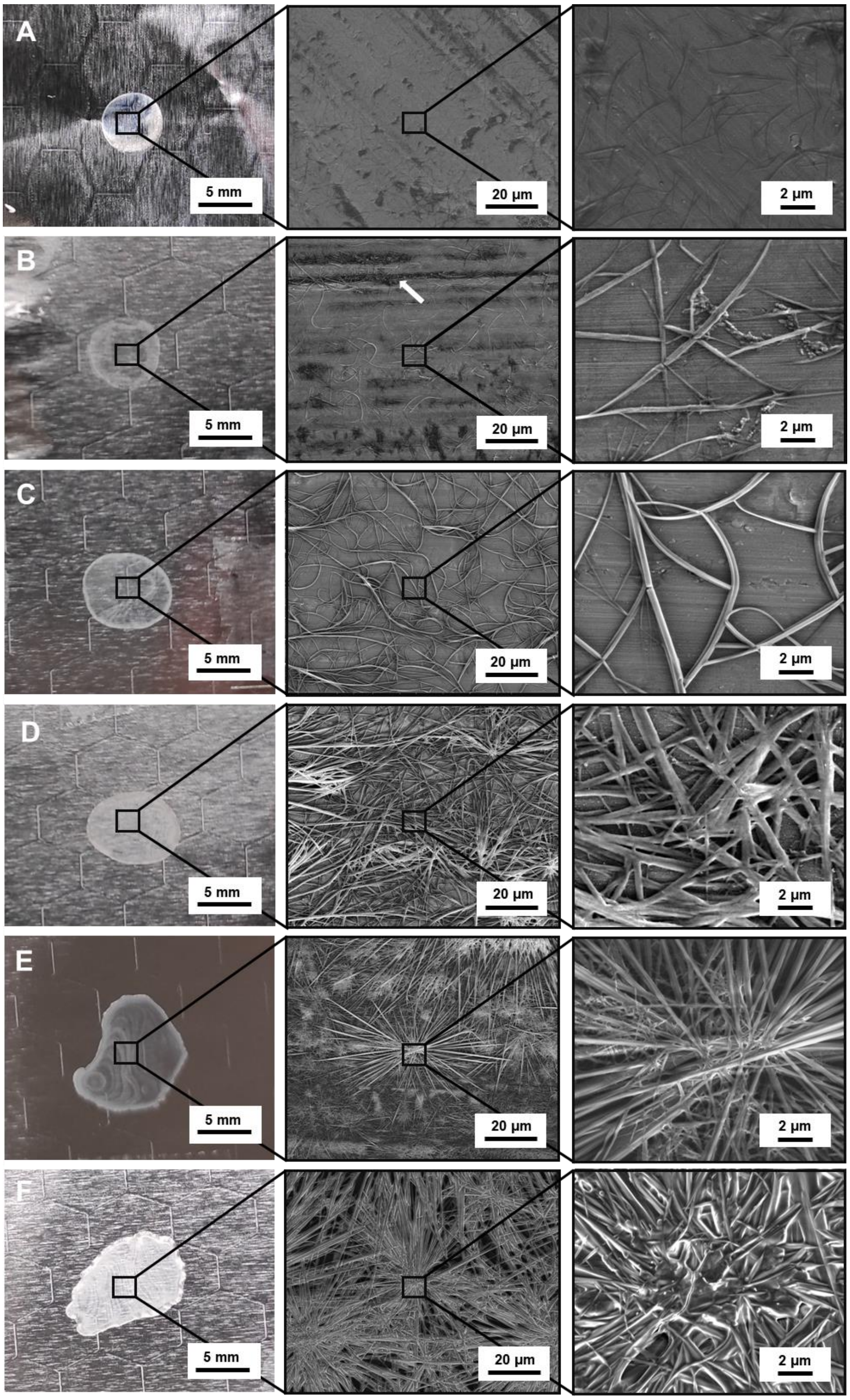

Figure S4. Optical (left) and scanning electron (middle and right with higher magnification) micrographs of the supramolecular fibres of BTA-Methyl prepared upon solvent evaporation from an A) 0.025 wt.% B) 0.050 wt.%, C) 0.100 wt.%, D) 0.250 wt.%, E) 0.500 wt.% and F) 1.000 wt.% aqueous solution onto aluminium foil.

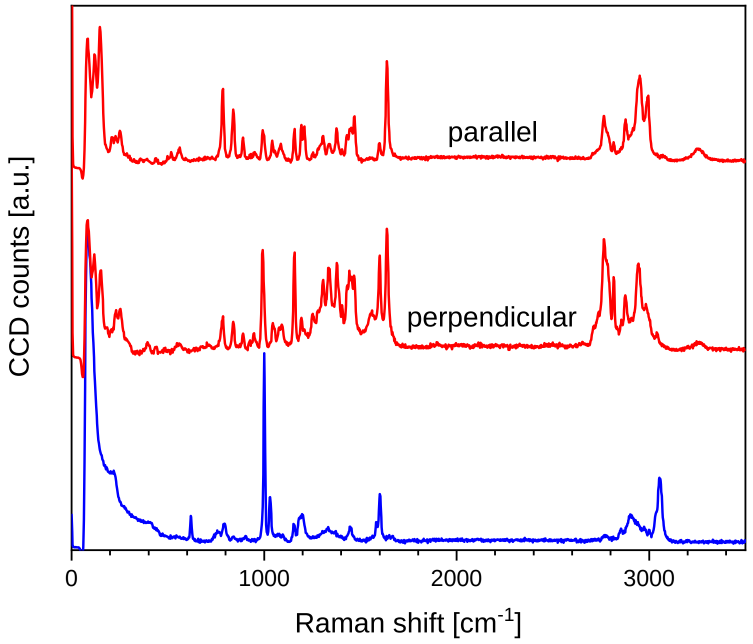

Figure S5. Raman measurements of polystyrene (blue) and a BTA-Methyl fibre (red) for polarization of the laser parallel and perpendicular to the self-assembled BTA-Methyl fibre long axis.

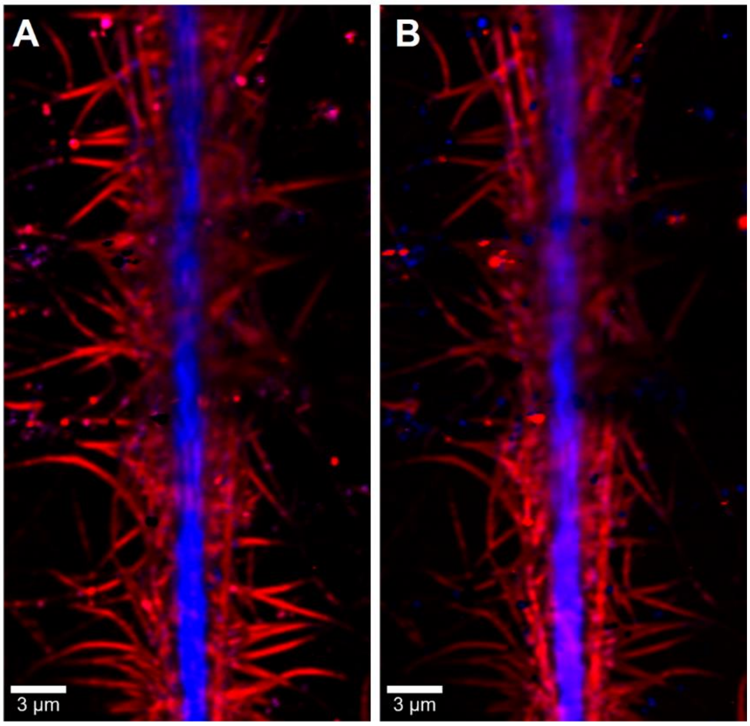

Figure S6. Spatially resolved component distribution from Raman imaging (horizontal polarization of the laser, PS rich regions are coloured in blue and BTA-Methyl rich in red), employing the Raman spectra of PS and BTA-Methyl for parallel and perpendicular orientation with respect to the laser polarization (Figure S5) in the true component analysis. As the Raman spectra of BTA-Methyl fibres depend on their orientation with respect to the laser polarization it is possible to extract the fraction of BTA-Methyl fibres being oriented parallel (A) and perpendicular (B) with respect to the laser, thus appearing more intense in the respective image.

References

[1] A. Frank, A. Bernet, K. Kreger, H.-W. Schmidt, *Soft Matter* **2020**, *16*, 4564.
[2] J. Schmelz, M. Karg, T. Hellweg, H. Schmalz, *ACS Nano* **2011**, *5*, 9523.
[3] C. Hils, M. Dulle, G. Sitaru, S. Gekle, J. Schöbel, A. Frank, M. Drechsler, A. Greiner, H. Schmalz, *Nanoscale Adv.* **2020**, *2*, 438.
[4] J. Schöbel, C. Hils, A. Weckwerth, M. Schlenk, C. Bojer, M. C. A. Stuart, J. Breu, S. Förster, A. Greiner, M. Karg, H. Schmalz, *Nanoscale* **2018**, *10*, 18257.
[5] J. Schmelz, D. Pirner, M. Krekhova, T. M. Ruhland, H. Schmalz, *Soft Matter* **2013**, *9*, 11173.
[6] J. Schöbel, M. Burgard, C. Hils, R. Dersch, M. Dulle, K. Volk, M. Karg, A. Greiner, H. Schmalz, *Angew. Chem., Int. Ed.* **2017**, *56*, 405; *Angew. Chem.* **2017**, *129*, 416.
[7] W.S., Rasband, ImageJ, U. S. National Institutes of Health, Bethesda, Maryland, USA, "https://imagej.nih.gov/ij/, 1997-2018".
